# Regulatory T cell therapy for xenotransplantation, what perspectives?

**DOI:** 10.3389/fimmu.2025.1685682

**Published:** 2025-09-19

**Authors:** Raphaël Porret, Erica Lana, Antonio Mancarella, Philippe Guillaume, Manuel Pascual, Raphael P. H. Meier, Jonathan S. Bromberg, Muhammad M. Mohiuddin, Leo H. Buhler, Qizhi Tang, Yannick D. Muller

**Affiliations:** ^1^ Division of Immunology and Allergy, Lausanne University Hospital and University of Lausanne, Lausanne, Switzerland; ^2^ Ludwig Institute for Cancer Research, Lausanne Branch, University of Lausanne, Lausanne, Switzerland; ^3^ Transplantation Center, Lausanne University Hospital and University of Lausanne, Lausanne, Switzerland; ^4^ University of Maryland School of Medicine, Department of Surgery, Baltimore, MD, United States; ^5^ Faculty of Science and Medicine, University of Fribourg, Fribourg, Switzerland; ^6^ Department of Surgery, University of California San Francisco, San Francisco, CA, United States; ^7^ Diabetes Center, University of California, San Francisco, San Francisco, CA, United States

**Keywords:** xenotransplantation, xenograft tolerance, regulatory T cells, genetic engineering, chimeric antigen receptor, cell therapy

## Abstract

Xenotransplantation has experienced major clinical advancements over the past three years. Yet, despite potent immunosuppressive regimens combining B-cell depleting therapies, T cell activation blockade, complement inhibition, and high-dose steroids, signs of antibody-mediated and cellular rejection were seen in the few pig-to human heart and kidney xenotransplants. Considering the recent success of chimeric antigen receptor T cell therapies in severe refractory autoimmune diseases, there are windows for opportunities to develop novel approaches to reduce the burden of immunosuppression. In this line, regulatory T cell (Treg) therapy is an attractive strategy, as Tregs could be genetically modified to recognize pig organs. In this brief review, we summarize the lessons learned from Tregs therapies in allotransplantation, update on the recent development in Treg research for xenotransplantation, and discuss future perspectives of humanizing pigs with human leukocyte antigens to promote tolerance using engineered Tregs.

## 1 Introduction

Xenotransplantation has experienced rapid advancements over the past three years, marked by reports of new clinical activities involving genetically engineered pig organs transplanted into deceased or live patients in the US and China (1). The results have shown survival or function up to 5 months, indicating that achieving greater control over the immune response will be essential for moving xenotransplantation into clinical practice (2–4). The use of CRISPR-Cas9 technologies has clearly increased the compatibility of pig organs for human transplantation (5). Recently, up to 69 genome edits were achieved in Yucatan miniature pigs. Types of edits included disrupting the glycan synthesis genes, inactivating the PERV elements and introducing a 7-transgene payload in the AAVS1 site that included *CD46* and *CD55* for the complement cascade, *THBD* and *PROCR* for the coagulation pathway, *CD47* don’t-eat-me molecule for restraining innate immune cells, and *TNFAIP3* and *HMOX1* to reduce ischemia-reperfusion injuries (6).

Yet, signs of antibody-mediated rejection was seen in the human cases of pig heart xenotransplants despite the use of a potent immunosuppressive regimen that included B-cell depleting therapies, T cell activation blockade, complement inhibition, and high-dose steroids (2, 7, 8). The recent case of a kidney-xenotransplant also showed early sign of cellular rejection that required treatment with steroids and thymoglobulin (3). Similarly, complement, IgM and IgG depositions were observed in the liver from a six-gene-edited pig which was transplanted into a brain-dead human (9). Finally, robust clonal expansion of CD8+ T cells and co-involvement of γδ T cells and NK cells were observed in a 61-day pig-to-human decedent thymokidney transplant (10). Altogether those results underscore the need for alternative immunosuppression regimen.

Complementary strategies beyond pig engineering should be explored to control rejection by favoring xenograft tolerance. Thymic transplantation and mixed hematopoietic chimerism targeting the central mechanism of immune tolerance have demonstrated promising outcomes in both preclinical models (11) and recently in phase 3 randomized clinical trial (12). Alternatively, regulatory T cell (Treg)-based therapies could represent a promising strategy to enhance peripheral tolerance in xenografts by modulating both humoral and cellular responses. Thus, their persistence in various xenograft models correlated with long-term graft survival (13, 14). Tregs are immune cells with dozens of built-in suppressive functions and can be categorized into different subsets, with the best characterized being thymic-derived Treg naturally expressing the transcription factor Forkhead box protein 3 (Foxp3). Importantly, Foxp3^+^ Tregs can be easily purified using cell-surface expression marker (CD4^+^CD127^low^CD25^high^), expanded ex vivo, and reinfused into patients (15).

This review aims to (1) summarize lessons learnt from Treg adoptive cell therapy (ACT) in allotransplantation (2), update on the recent development in Tregs research for xenotransplantation (3), discuss the rationale for humanizing pig with human leukocyte antigen (HLA) to redirect the specificity of next-generation Tregs therapies for xenotransplantation and to improve their efficacy.

## 2 Methods

The following terms were used on ClinicalTrials.gov: Transplantation-OR-Solid organ transplant-OR-xenotransplant-AND-regulatory t cell therapy-OR-Treg therapy-OR-T-regulatory cells-NOT-GVHD-NOT-graft-versus-host-NOT tumor We identified 53 trials, of which 26 were selected. One additional trial (NCT04950842) was included based on a published study (16). Two trials (ISRCTN11038572, ISRCTN15374803) were included from the ISRCTN registry, and one from the UMIN-CTR registry (UMIN000015789) (17). Cell therapy products enriched in Tregs (e.g., peripheral blood mononuclear cells stimulated with donor cells in the presence of co-stimulation blockade) were not included in this review. Regarding the literature on Treg therapy in xenotransplantation, a PubMed search (2010–2025) using terms related to Tregs and xenotransplantation, excluding oncology-related keywords, retrieved 22 articles. Eight research articles directly addressing Treg therapy in xenotransplantation were selected.

## 3 Lessons learnt from allotransplantation

**Figure 1:**
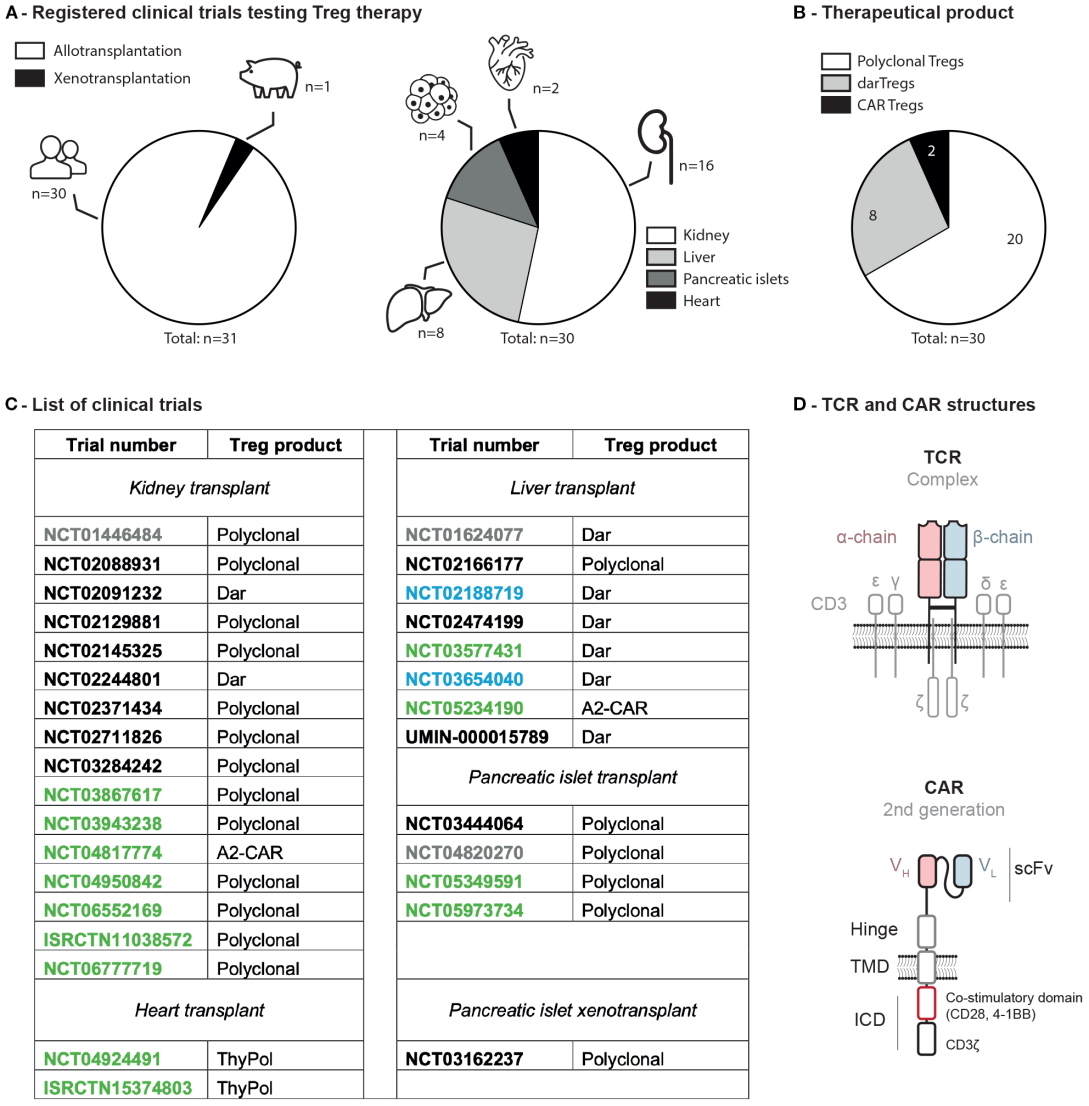
Clinical trials assessing Tregs to induce donor-specific organ tolerance in SOT. **(A)** Registered trials in allo- versus xeno-transplantation and organs associated. **(B)** Type of Treg infused. **(C)** List of clinical trials involving Treg infusion in solid organ transplantation. Status as of 2025-06-26: green, active; black, completed; blue, terminated; grey, unknown. **(D)** Molecular structures of a TCR and a CAR. ICD, intracellular domain; scFv, small chain variable fragment; TMD, transmembrane domain; VH, variable heavy; VL, variable light; A2-CAR, anti-HLA-A2 chimeric antigen receptor; dar, donor alloantigen-reactive; ISRCTN, international standard randomized controlled trial number; NCT, national clinical trial; SOT, solid organ transplant; TCR, T cell receptor; ThyPol, polyclonal from thymus; Treg, regulatory T cell.

Before designing a Treg therapy for xenotransplantation, it is essential to consider the lessons already learned in allotransplantation. Thus, as of June 26^th^, 2025, 30 trials have been registered: sixteen involving kidney, eight liver, four pancreatic islet, and two heart transplant recipients (**Figure 1A**). Therapeutic products included polyclonal (i.e. nonspecifically expanded) Tregs (twenty trials), donor alloantigen-reactive (dar, i.e. expanded in the presence of donor derived B cells) Tregs (eight trials), and chimeric antigen receptor (CAR, i.e. engineered with a viral vector) Tregs (two trials) (**Figures 1B, C**). Of note, one clinical trial (NCT03162237) testing polyclonal Tregs for porcine islets xenotransplantation was initiated in 2013 in China.

### 3.1 Autologous Treg injection is feasible and safe

Several studies have confirmed the feasibility and safety of autologous Treg therapies. In the ONE study, a total of 28 living-donor kidney transplant patients received an autologous Treg infusion which were either polyspecific (stimulated nonspecifically with anti-CD3/CD28) or donor alloreactive (dar; i.e. stimulated/expanded with donor-derived antigen presenting cells). Both type of Tregs infusion were well tolerated and considered safe (18–20). In kidney transplantation, two other studies confirmed the safety and the feasibility of this approach: the TRACT trial (NCT02145325) (21), and the TASK study (NCT02088931) (22). This was also corroborated in liver transplantation by the ThRIL trial (NCT02166177) (23) and the ARTEMIS trial (NCT02474199) (24). The Spanish THYTECH trial (NCT04924491) evaluated Tregs isolated from thymic tissue (thyTreg) for heart transplantation in children. Thus, the thymus is routinely removed during pediatric cardiac surgery representing an important source of Tregs (25). Across all reports, Tregs did not affect negatively organ function confirming that this therapy can be considered for xenotransplantation.

### 3.2 Persistence of autologous Tregs

A concern with Tregs therapy is related to their limited persistence in time, as most cells are lost after infusion. In the TASK study (NCT02088931), deuterated glucose labeling showed that infused Tregs persisted at similar levels regardless of the type of immunosuppression, representing 2-8% of circulating Tregs in the first week and 0.2% at three months post-infusion. No deuterium was detected in non-Treg subsets, indicating lineage stability. Kidney biopsies at two weeks and six months showed that 0.5–5.9% of infiltrating CD4^+^ T cells expressed Foxp3, but only 0.2% carried a deuterium label (22). In the ARTEMIS trial (NCT02474199), deuterated-labeled darTreg persistence correlated with dose, ranging from 0.25-2% of circulating Tregs on infusion day to 0.25-0.5% beyond 300 days post-transplant (24). In contrast, the TRACT and ThRIL trials did not label infused Tregs. Still, TRACT showed a sustained 5- to 20-fold increase in circulating Tregs throughout the one-year follow-up period (21), while ThRIL reported a transient 1.2-1.5-fold increase only during the first month post-infusion of the higher dose (4.5x10^6^ Tregs/kg) (23). Altogether, even if a fraction of Tregs can persist in chronically immunosuppressed patients, repetitive Tregs infusion may be necessary for xenotransplantation.

### 3.3 Treg manufacturing, a challenge

The manufacturing of Tregs remain difficult for two main reasons. First, the prolonged immunosuppressive regimen impair the cell’s fitness limiting their expansion capabilities ex vivo (24). Secondly, darTregs may also leave the circulation after transplantation reducing the pool of circulating allogenic cells when starting the Treg isolation (24). In the ARTEMIS trial (NCT02474199), four of nine expansions failed to meet the minimal infusible dose, which was attributed to the low Treg counts in the peripheral blood of liver transplant recipients (24). The deLTA trial (NCT02188719) was terminated prematurely, in part due to manufacturing difficulties. In the ONE study (NCT02129881) four preparations could not be dosed, three because of insufficient cell numbers and one because of bacterial contamination. High levels of variability were observed in the expansion capacity of the cells *in vitro* (24). In the ThRIL trial (NCT02166177), two of eleven Treg expansions failed, one likely due to a low starting Treg count, and the other due to insufficient purity of the final product (46% CD4^+^CD25^+^Foxp3^+^ cells) (23). Overall, allogenic sources (e.g., from the thymus or cord blood) of Tregs may become preferable for xenotransplantation in particular if multiple infusions are envisioned.

### 3.4 Efficacy of polyclonal and donor-reactive Tregs

The best proof of principle of clinical efficacy has been observed in prevention of graft versus host disease after stem cell transplantation (26). In organ transplantation, despite the large number of registered trials, Treg therapy has shown less impressive results so far. In the ONE study, 52.2% of patients given polyclonal Tregs were successfully weaned from mycophenolate mofetil and maintained on tacrolimus monotherapy. Even if the rate biopsy-confirmed acute rejection remained stable, the risk of infection was significantly reduced as compared to historical controls (18–20). The phase IIb TWO study (ISRCTN11038572), currently recruiting in the United Kingdom, aims to validate the efficacy of polyclonal Tregs by assessing its impact on biopsy-proven acute rejection and on lessening the immunosuppression burden. In the ARTEMIS trial, the primary efficacy endpoint was calcineurin inhibitor dose reduction by 75% with stable liver function tests for at least 12 weeks (24). Among the five patients who could be treated with darTregs, two reached the primary end point; a number too small to conclude on efficacy. Therefore, one could expect a limited benefit from a polyclonal or xenoreactive Treg products, which should encourage to engineer Treg with synthetic receptors.

### 3.5 Anti-HLA-A2 CAR Tregs in clinical trials

Among the different strategies employed to enrich for Treg alloreactivity, the most efficient modality in preclinical models is to use CAR that recognizes an HLA molecule only expressed in the transplanted organ. Considering the high prevalence of HLA-A2 in the general population (27), the most studied CAR is directed against HLA-A*02:01 (A2). Thus, in preclinical models, A2-CAR Tregs have shown superior efficacy over other modalities as they gain enhanced trafficking and suppressive activities in GvHD but also skin, islets and heart transplant models (16, 28–31). These results led to the initiation of two clinical trials testing A2-CAR Tregs in HLA-A2 negative recipients transplanted with either kidney (STEADFAST, NCT04817774) or liver (LIBERATE, NCT05234190) from HLA-A2 positive donors (**Figure 1B**). A few patients were already transplanted and dosed with A2-CAR Tregs. A2-CAR Tregs were found in the liver biopsy at 1 month post-treatment and no treatment-related adverse events were reported (32).

## 4 Tregs in xenotransplantation: what’s new?

Since our first reviews of early studies demonstrating the feasibility of expanding xenogeneic-specific Tregs, achieving up to 3500 fold expansion (33), a few more reports have been published although the overall literature in this field remains sparse. *In vitro*, xenoantigen-stimulated Tregs displayed enhanced suppressive function compared to their polyclonal counterparts in a xenogeneic mixed lymphocyte reaction (MLR) (34). Jin et al. confirmed that porcine-reactive human Tregs were more suppressive than polyclonal Tregs in protecting neonatal porcine islet cell clusters (NICCs) with a survival beyond 84 days compared to 63 days with unspecific polyclonal Tregs (35). Not surprisingly, CD27^+^, an activation and memory Treg marker was associated with higher expression of Foxp3, cytotoxic T-lymphocyte antigen 4 (CTLA-4), and Helios expression correlating with >60 days protection in porcine skin xenograft mouse model (36). Duong et al. showed that long-term xenograft survival correlated with CD39 expression in baboon Tregs representing another potential biomarker for identifying Foxp3^high^ suppressive Tregs (37). Importantly, IL-10 produced by human Tregs was critical for suppressing xenogeneic effector T cell proliferation *in vitro* (38), and for prolonging NICC survival *in vivo*, as blocking this cytokine with an anti-human IL-10 monoclonal antibody significantly shortened the xenograft survival (39).

In non-human primates (NHP) models, five monkeys were transplanted with pig islet xenografts, all treated with cobra venom factor (to deplete complement), anti-thymocyte globulin for induction therapy, and with anti-CD154 monoclonal antibody and low-dose sirolimus for maintenance therapy. Three NHPs were infused with autologous polyclonal Tregs at the peri-transplantation period. Of the five NHPs, two remained insulin-independent for > 500 days, and both had received Tregs (40). Yet, upon discontinuing immunosuppression, the two NHPs did not maintain tolerance (41). In those animals the autoantibody titers correlated with a loss of glycemic control and a dense CD4^+^ and CD8^+^ T cell infiltration in the islets biopsies, indicating that adoptively transferred Tregs were insufficient to induce durable transplant tolerance (41). Whether repetitive infusion of Tregs after immunosuppression withdrawal could lead to better outcomes remains to be defined. Interestingly, the persistence of detectable circulating CD4^+^CD25^high^Foxp3^+^ correlated with the long-term survival of pig-to-NHP heart xenotransplants (13). In this model, Treg levels remained elevated until rejection and could, therefore, also represent a biomarker for monitoring the graft survival (13).

## 5 Next-generation xenograft-specific Tregs

Recent advances in gene editing for T cells open new opportunities to develop next-generation Treg therapies for xenotransplantation (15). Circulating antigen-specific Tregs are rare and difficult to expand from the peripheral blood in immunosuppressed patients. Redirecting the specificity of Tregs with synthetic genes using lentiviral or retroviral vectors could circumvent these limitations (15). In this regard, two types of receptors can be engineered: T cell receptor (TCR) and CAR. While TCRs are physiological receptors, CARs are artificial immune transmembrane receptors composed of an antigen-binding domain, usually a single-chain variable fragment from an antibody (scFv), a hinge, a transmembrane domain, and an intracellular signaling domain (**Figure 1D**) (15). The main advantage of CARs over TCRs is their ability to recognize any cell surface, matrix, or multivalent soluble antigen. In contrast, TCRs target major histocompatibility complex known as human leukocyte antigen in humans (HLA) and swine leukocyte antigen (SLA) in pigs. While no direct comparison exists between TCR- and CAR-engineered Tregs, a recent study indicated a significant survival advantage for the A2-CAR Treg-treated group compared to those receiving polyclonal or darTregs in a GvHD mouse model (16). Thus, CARs constitute an attractive way to redirect T cells against a wider array of tissue- or cell-specific antigen. Both TCRs and CARs can recognize the HLA or SLA system involved in antigen presentation and immune recognition and therefore can be used to arm Tregs in xenotransplantation.

## 6 Perspectives for Treg therapy in xenotransplantation

**Figure 2:**
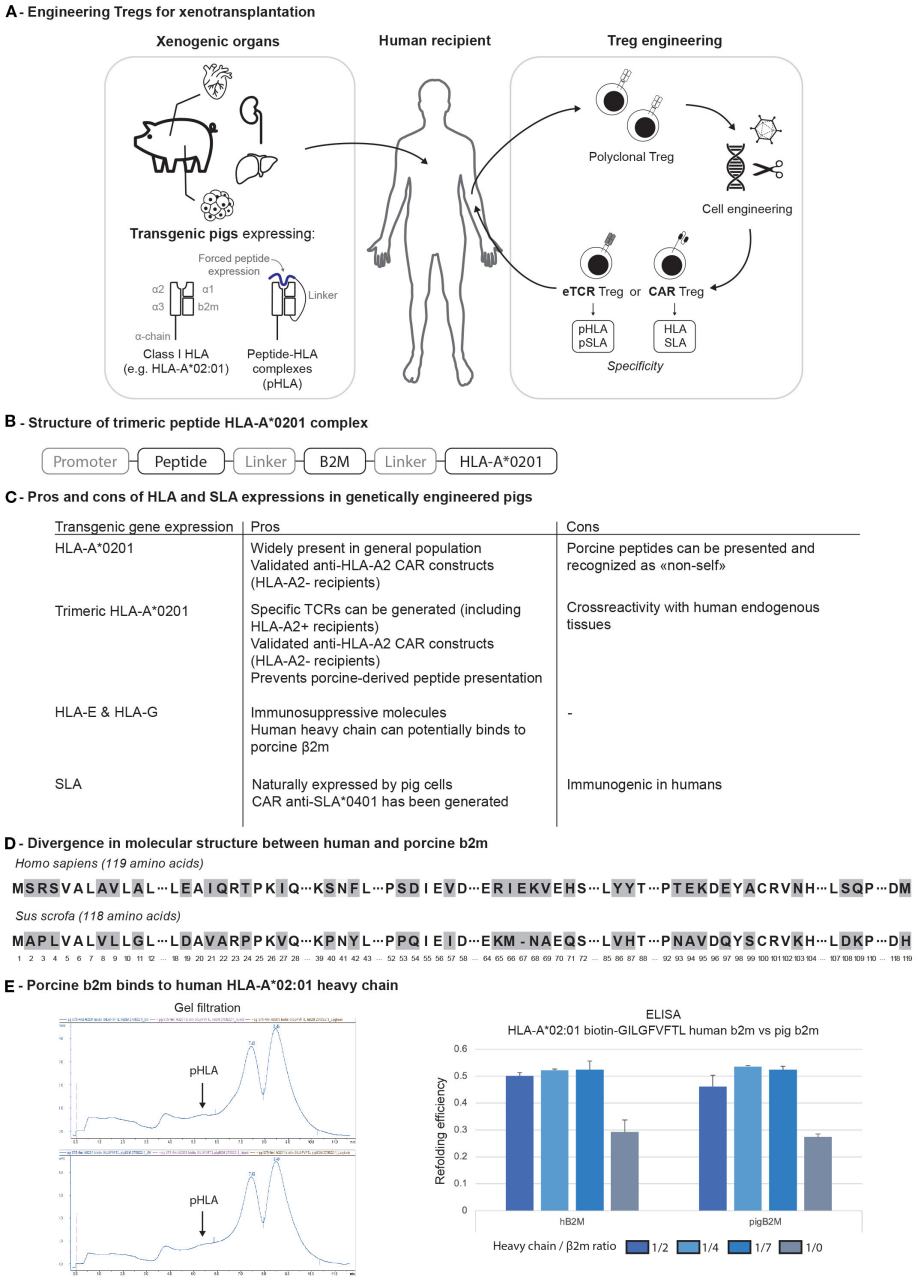
**(A)** Engineering of both pigs and Tregs for xenotransplantation. **(B)** Trimeric structure of pHLA complex. **(C)** Pros and cons of transgenic major histocompatibility complex molecule expression on genetically engineered pigs. **(D)** Divergence in molecular structure between human and porcine β2m. **(E)** Binding of human and pig b2m to HLA-A*02:01 heavy chain. b2m, b2-microglobulin; CAR, chimeric antigen receptor; HLA, human leukocyte antigen; eTCR, engineered T cell receptor; pHLA, peptide-HLA complex.

In xenotransplantation, as both the recipient’s cells and the pig donor can be genetically modified, several approaches can be envisioned, either by redirecting human Tregs against SLA using existing pigs or by further modifying pigs to express HLAs (**Figure 2A**). At the International Congress of the Transplant Society in 2022, anti-SLA*0401 CAR Treg protection of porcine skin and pancreatic islet xenografts in humanized mouse model was reported (42). Another strategy would be to generate HLA-A2 transgenic pig cells. This would have the main advantage of being compatible with clinically tested A2-CAR Treg. Yet, only HLA-A2 negative recipients could be treated using this approach. In addition, expressing an HLA on porcine tissue may also increase presentation of porcine peptides thus increase the immunogenicity and risk of rejection.

To address both issues, one could engineer pigs with a peptide-HLA (pHLA) trimeric complex (**Figure 2B**). In this configuration, selecting an immunodominant peptide naturally presented in the thymus (e.g., derived from the insulin) would (1) prevent the risk of developing an auto-”xeno”-reactive repertoire of T cells due to the thymic negative selection (2), block the presentation of xenogeneic peptide on HLA-A2 molecules in the xenograft (3), engineer specific TCRs or CARs (**Figure 2C**). The same pig could be used for CAR Treg therapy in A2-negative recipients and TCR-engineered (eTCR) Treg therapy in A2-positive patients (**Figure 2C**).

An alternative strategy could involve the engineering of xeno-reactive Tregs (against SLA) (43). Unfortunately, to the best of our knowledge, SLA-specific human TCRs are not publicly available. This could be addressed through high-throughput single-cell TCR sequencing from the recently transplanted patients who rejected their graft (2, 44). Alternatively, expanding SLA-reactive Tregs in cell cultures may also be a source of materials for TCR discovery. In any case, when engineering a TCR, there is an important risk for TCR mispairing with the endogenous chains. Such concern will require base-editing CRISPR technologies reviewed elsewhere (15).

### 6.1 Removing SLA to create a favorable environment for Treg suppression

To increase the immunosurveillance capacities of Tregs, it would still be important to reduce the pig immunogenicity by reducing SLA class I expression and/or removing SLA class II. Yet, complete *B2M* deletion shortens pig lifespan, with affected animals developing fever around four weeks of age and succumbing to septicemia, as evidenced by multiorgan lymph node enlargement and bacterial infiltrates observed during autopsy (45). To address this concern, triple modified pigs (*B2M*, *GGTA1*, *CMAH*) were generated in Germany with an SLA class I^low^ phenotype, introducing only a partial deletion of the *B2M* gene. These animals remained viable and displayed reduced immunogenicity, as indicated by lower proliferation of human peripheral blood mononuclear cells (PBMCs) *in vitro* (46). Such approach may remain limited if soluble human b2m would restore some SLA expression considering the close homology with pig b2m (**Figures 2D, E**). The functional consequences of *B2M* KO on iron homeostasis may be another limitation (47).

Beyond *B2M* KO pigs, SLA class II-deficient pigs were also successfully generated by targeting the *CIITA* gene. Four-gene KO (*GGTA1*, *CMAH*, *β4GalNT2*, *CIITA*) pigs survived for over a year and displayed reduced CD4^+^ T cell proliferation in mixed lymphocyte reactions (48). Class I and class II SLA-deficient pigs were also generated (49, 50). Consistent with previous reports (45), animal survival was compromised which requires decontaminated facilities and high-standard procedures such as cesarean delivery and breeding. Thus, missing SLAs impairs the fitness of T and NK cells.

## 7 Conclusion

Transgenic pigs have been successfully engineered to downregulate the immune response towards xenografts. They could be further humanized and express HLA molecules. This would enable the specific recruitment of engineered CAR or TCR Tregs which would be transgene specific. By forcing the presentation of self-derived peptides, we could further prevent the risk of presenting porcine-derived peptides. In this perspective, HLA-A2 may be a great candidate due to its high prevalence in the general population and because of the numerous pre-clinical results with A2-CAR Tregs. Expressing additional HLA-E and HLA-G molecules, known to bind to inhibitory receptors expressed on natural killer (NK) and T cells, could further contribute to establish xenograft tolerance. Engineering those HLAs in animals who are already SLA class I deficient, alternatively *CIITA* KO SLA class II-deficient, may be the most efficient strategy to best protect the xenograft in the future.

While Treg therapy is still under evaluation in allotransplantation, the unique immunological context of xenotransplantation presents an opportunity to study Tregs’ ability to home to the graft ultimately reducing the burden of immunosuppression. Their efficacy should be confirmed in NHP xenotransplantation models, including the use of repeated infusions, ideally from allogeneic sources.
